# Modeling of Cell Nuclear Mechanics: Classes, Components, and Applications

**DOI:** 10.3390/cells9071623

**Published:** 2020-07-06

**Authors:** Chad M. Hobson, Andrew D. Stephens

**Affiliations:** 1 Department of Physics and Astronomy, The University of North Carolina at Chapel Hill, Chapel Hill, NC 27599, USA; 2 Biology Department, The University of Massachusetts at Amherst, Amherst, MA 01003, USA

**Keywords:** nuclear mechanics, modeling, lamins, chromatin, cytoskeleton, continuum, discrete

## Abstract

Cell nuclei are paramount for both cellular function and mechanical stability. These two roles of nuclei are intertwined as altered mechanical properties of nuclei are associated with altered cell behavior and disease. To further understand the mechanical properties of cell nuclei and guide future experiments, many investigators have turned to mechanical modeling. Here, we provide a comprehensive review of mechanical modeling of cell nuclei with an emphasis on the role of the nuclear lamina in hopes of spurring future growth of this field. The goal of this review is to provide an introduction to mechanical modeling techniques, highlight current applications to nuclear mechanics, and give insight into future directions of mechanical modeling. There are three main classes of mechanical models—schematic, continuum mechanics, and molecular dynamics—which provide unique advantages and limitations. Current experimental understanding of the roles of the cytoskeleton, the nuclear lamina, and the chromatin in nuclear mechanics provide the basis for how each component is subsequently treated in mechanical models. Modeling allows us to interpret assay-specific experimental results for key parameters and quantitatively predict emergent behaviors. This is specifically powerful when emergent phenomena, such as lamin-based strain stiffening, can be deduced from complimentary experimental techniques. Modeling differences in force application, geometry, or composition can additionally clarify seemingly conflicting experimental results. Using these approaches, mechanical models have informed our understanding of relevant biological processes such as migration, nuclear blebbing, nuclear rupture, and cell spreading and detachment. There remain many aspects of nuclear mechanics for which additional mechanical modeling could provide immediate insight. Although mechanical modeling of cell nuclei has been employed for over a decade, there are still relatively few models for any given biological phenomenon. This implies that an influx of research into this realm of the field has the potential to dramatically shape both future experiments and our current understanding of nuclear mechanics, function, and disease.

## 1. Introduction

The cell nucleus is not only the site of transcriptional activity and DNA replication in eukaryotic cells, but its mechanical properties additionally serve to both protect the genome and transfer mechanical signals from the extracellular environment to the chromatin. The nuclear lamina, which forms a protein meshwork along the underside of the nuclear envelope, is one of the primary mechanical constituents of the nucleus. A variety of disease states ranging from heart disease [1] to premature aging called progeria [2] are associated with defects in the nuclear lamina. These and many other diseases, such as cancer [3,4,5,6], are known to be associated with both altered nuclear morphology and mechanical properties, which are then known to increase nuclear blebbing, nuclear rupture, DNA damage, and cellular invasiveness. Mechanical modelling of cell nuclei has become a growing area of research for the last decade because of the physical nature of these processes. The purpose of this article is to introduce mechanical modeling to a broad audience as well as discuss a variety of current and future applications of mechanical modeling regarding cell nuclei. In this review, we outline the primary classes of mechanical models along with their advantages and limitations. Additionally, we detail the current experimental understanding of the mechanical constituents of the nucleus, including the cytoskeleton, lamins, and chromatin, as well as the ways in which they have been modeled. Subsequently, we discuss the importance of mechanical models in interpreting experimental data across different assays. Finally, we detail how mechanical models have been used to further our collective understanding of biologically relevant processes such as nuclear blebbing and rupture, as well as lay out areas of nuclear mechanics in need of additional modeling. Given the extensive role of the nucleus in cellular function and the association of mutations in the genes that encode the nuclear lamins with disease, mechanical modeling has and will continue to serve a vital role in interpreting current results and providing predictions to guide new experiments.

## 2. Classifications of Mechanical Models

**Figure 1:**
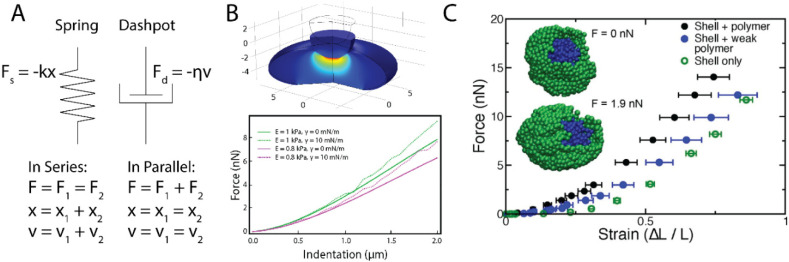
(**A**) Common components for 1D viscoelastic schematic models where F is the force across a given element, k is the spring constant, η is the viscosity, x is displacement, and v is velocity. (**B**) A continuum mechanics (CM) model of atomic force microscopy where the nucleus is treated as an elastic solid surrounded by a thin, elastic shell. The axes represent distance in μm. Force versus indentation data show strain stiffening during compression where the small indentation regime is dictated by the elastic solid and the large indentation regime is dominated by stretching of the elastic shell. Reprinted with permission from Hobson et al. (2020) [7]. (**C**) A molecular dynamics (MD) simulation of micromanipulation of an isolated nucleus. The nucleus is modeled to have a crosslinked polymeric interior that is linked to a polymeric shell. The presence of the polymer interior dictates the initial force response while the shell results in a strain-stiffening response during long extension. Reprinted with permission from Stephens et al. (2017) [8].

Broadly speaking, mechanical models of nuclei can be broken down into three categories: (i) schematic models, (ii) continuum mechanics (CM) models, and (iii) molecular dynamics (MD) simulations (Figure 1). Schematic models are not necessarily specific to nuclei as they are often unable to account for geometry and separate components, whereas CM and MD models are designed to be nucleus-specific with accurate geometries and separate roles of nuclear constituents. Other approaches and techniques exist, but a majority of the mechanical models of cell nuclei fall into the aforementioned three categories. Each of these classes have a unique set of advantages and limitations, as reviewed below and summarized in Table 1. One model type is not necessarily better than another, but rather different. The level of specificity and type of a model should be dictated by the phenomena being studied.

### 2.1. Schematic Models

Schematic models are those which use simple combinations of springs and dashpots to provide one-dimensional relationships between stress, strain, and time. A spring has a force response that is proportional to extension or compression while a dashpot has a force response that is proportional to the rate of extension or compression (Figure 1A). The primary benefit of schematic models is that, once solved, they provide a set of exact equations to be fit to a given dataset. Because of their inherent simplicity, schematic models are extremely successful in detecting changes in mechanical properties due to biological intervention. A classic example is the Jeffreys model which features a spring and a dashpot in parallel, together in series with a second dashpot. This model, when applied to micropipette aspiration data collected on MEFs, has been used to show how lamin A/C-deficient nuclei have reduced viscosity and elasticity [9]. Similarly, a standard linear solid model which features a spring and dashpot in series together in parallel with a second spring has been used to show isolated chondrocyte nuclei are stiffer and more viscous than intact chondrocytes [10]. Schematic models are also quite successful at studying limiting cases: that is, cases where certain structures or phenomena dominate the entire system, allowing for the system’s response to be distilled into simple elements such as springs and dashpots. The primary advantages of schematic models are then their ease of use and accessibility, their ability to detect changes in mechanical properties under different conditions, and their success in studying limiting cases.

Schematic models also have a suite of limitations when applied to nuclear mechanics. Exact solutions to physical problems characteristic of these models typically require idealized conditions and sweeping assumptions that almost certainly do not hold true for biophysical problems. Cell nuclei have been shown to be anisotropic [11], heterogeneous [12], and strain stiffening in both compression [7] and extension [8]; this directly invalidates these key assumptions. One must then be cautious in interpreting the absolute magnitude of the mechanical properties determined by such schematic models as their assumptions are often not met. Other common assumptions surround nuclear geometry and structure, where in schematic models the nucleus is often assumed to be a one-dimensional object that glosses over the intricacies of the chromatin, lamins, and their connections. Revisiting the aforementioned example of the Jeffreys model, the investigators showed a significant decrease in all parameters associated with the model when comparing lamin A/C-deficient MEFs to healthy MEFs. This means the model was not successful in distinguishing the specific role of lamin A/C in nuclear mechanics as it could not be associated with a specific element of the model being applied. Simply put, schematic models make it difficult to determine differential contributions of nuclear components. Along these lines, schematic models are generally not specific to cell nuclei; that is, each element in the model is not necessarily equated to a mechanical constituent of the nucleus. Schematic models are rather just applied to data taken on cell nuclei. Schematic models provide an approachable and easy-to-use means of characterizing changes in mechanical properties and studying limiting cases or dominant phenomena. However, users must be wary of the assumptions made in their derivation when considering their results.

### 2.2. Continuum Mechanics (CM) Models

CM models are those which assume that the materials being modeled are continuous as opposed to sets of discretized particles (Figure 1B). These models can either be solved analytically or computationally. The former generally requires more stringent assumptions while the latter allows for additional flexibility and specificity. A classic example of an analytically solved CM model is the Hertz contact mechanics model which presumes contact between two linearly elastic, homogeneous, isotropic solids under small indentations [13], which is an idealistic approximation of a nucleus under compression. The Hertz model has been used extensively and successfully in atomic force microscopy (AFM) studies to highlight the importance of chromatin compaction and lamin A/C in the mechanical integrity of nuclei [14,15]. However, computationally solving CM models through means such as finite element analysis (FEA) allows investigators to circumvent some of assumptions of analytically-solved CM models at the cost of computational intensity. For example, computationally solved CM models of AFM have allowed investigators to move beyond the Hertz model and study the separate roles of lamins and chromatin, more accurate nuclear geometries, and viscous contributions [7,16,17,18]. The first benefit of this approach is in the flexibility of the geometry of the system. The investigator can not only set up the model to more accurately describe the geometry of the nucleus being studied, but also vary this geometry to understand the dependence of the mechanical response on the geometry itself. An example is one of the foundation studies of nuclear mechanics which modeled plate compression of spread, rounded, and isolated nuclei [19]. Furthermore, an additional advantage is the ability to prescribe layers to a system, each with different mechanical properties. This is a simple way to model, for example, the inner and outer nuclear membranes as well as the nuclear lamina separately with little added complexity for the user [16,20]. Additionally, each of these layers can be prescribed a separate constitutive law based on the current understanding of the literature. Finally, CM models are quite advantageous for modeling specific assays because of their ability to quickly and precisely set up the geometry of a system. 

There are limitations, however, to using CM models. The first of these is that CM models assume that each material and layer is continuous. More specifically, CM models do not model polymeric systems or motion of individual proteins, but instead smooth over the complex filamentous nature of, for example, the nuclear lamina and treats it as a continuous solid shell. This is an inherent limitation in that CM models do not account for the true physical structure of the constituents of the nucleus, such as the meshwork nature of the nuclear lamina [21,22,23]. Furthermore, CM models require that you know and prescribe the mechanical properties of each material a priori. This is in contrast to a simulation for which the mechanical properties are emergent. CM models are then advantageous for prescribing realistic geometries and easily modeling multiple layers with different material properties. However, they are limited in that they require a priori assumptions of the material properties of the system and cannot model non-continuous materials.

### 2.3. Molecular Dynamics (MD)

The final class of models are MD simulations (Figure 1C). In these MD simulations, nuclei are discretized on a quasi-microscopic level. Individual molecules can be linked together through specified interactions—such as a spring-like force—to form polymeric chains with motion subject to Newton’s laws. The polymers can then be organized to form structures such as chromatin fibers or the nuclear lamina, and subsequently be subjected to external forces. The first advantage of MD simulations is that they can provide a more accurate structural representation as both chromatin and the nuclear lamina are effectively polymer networks [21,23,24,25]. Furthermore, these models allow the investigator to prescribe both the location and strength of the bonds between nuclear substructures. For example, one recent study has investigated the role of the bond strength between the cytoskeleton and the nucleus in regulating nuclear shape fluctuations [26]. A third unique advantage of MD simulations is that the mechanical response is often emergent and not prescribed. That is, the investigator will define the specific interactions between monomers to form polymers as well as the number of polymers and the interactions between them, but the simulation will then reveal the mechanical response of this polymer meshwork. This has been used to show that nuclei exhibit strain stiffening in micromanipulation experiments without needing to prescribe any strain-stiffening phenomena directly into the model itself [8,27]. Similar to CM models, MD simulations allow investigators to easily prescribe multiple materials with varied interactions. 

As with the previous techniques, MD simulations also have a set of limitations. First, MD simulations are limited in their accessibility to the broader biological community. They often require extensive computational power and expertise to design effective and realistic MD simulations. An additional limitation is that they are often coarse-grained simulations. That is, there is often a loss of structural detail in both the chromatin and lamin networks. Coarse-graining the model then means that tuning the strength and number of interactions between monomers is non-trivial and not necessarily indicative of the true molecular-scale interactions. They are also limited in their ability to study how varying the material properties of a specific nuclear substructure alters the mechanical response. In an MD simulation, one can alter that interactions between monomers, but not specifically change the overall material properties as easily as in CM models. MD simulations then provide a means of modeling the polymeric nature of the nuclear substructures as well as allow for emergent phenomena to be discovered, but they are limited in overall accessibility and the need for precise knowledge of quasi-molecular-scale interactions.

## 3. Nuclear Mechanical Constituents and How They Are Modeled

**Figure 2:**
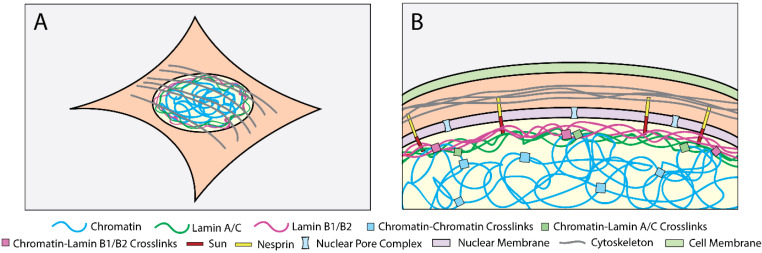
(**A**) A schematic of a whole cell. (**B**) A close-up, cross-section schematic of the ventral half of the cell drawn in (**A**). The proteins, structures, and interactions are a subset of the full system, but are historically those which are relevant to consider when developing a mechanical model for nuclei.

While the cell nucleus is a beautifully complex system, its mechanical response is in general dependent on three things: the cytoskeleton, the nuclear lamina, and the chromatin (Figure 2). These structures are themselves composed of multiple constituents, each of which are dynamic with intricate molecular-scale interactions [28,29,30]. Additionally, these structures are not independent. They form a mechanical and biochemical pathway from integrin receptors to the nuclear interior, capable of propagating forces on the cell surface to the DNA and subsequently altering transcription [31,32,33]. Mechanical modeling, however, has not yet reached this full level of detail and often takes a coarse-grained approach to studying their respective roles. Here, we review the current understanding of the mechanical roles of these three structures based on experimental data and how they have subsequently been treated in nucleus-specific CM models and MD simulations (Table 2) [34].

### 3.1. Cytoskeleton

The cytoskeleton primarily consists of actin, microtubules, and intermediate filaments. Arguably the most important of these constituents for nuclear integrity and mechanics are actin and vimentin intermediate filaments. In spread cells, actin has been experimentally shown to form a perinuclear cap that is important for mechanosensation, cell migration, and nuclear shape [50,51,52,53]. Additionally, Arp2/3-driven actin polymerization has been shown to disrupt the nuclear lamina and facilitate constricted migration [54]. The cytoskeleton also serves an antagonistic rule as both actin and microtubules can deform the nucleus [55,56,57]. The literature regarding actin and the actual mechanical properties of the nucleus are seemingly conflicting, likely due to extension- versus compression-based force measurements and the aforementioned antagonistic behavior. Single and dual micromanipulation extension studies have concluded that actin is not critical for protecting against nuclear shape change under external force [58] or in vivo or ex vivo single nucleus force measurements [8]. AFM studies with sharp probes compressing nuclei, however, concluded actin depolymerization reduces nuclear elasticity and viscosity [59]. Vimentin—an intermediate filament shown to be itself strain stiffening [60,61]—forms a perinuclear cage [62]. This cage has been experimentally shown to maintain nuclear positioning and deformation in the cell through single micromanipulation studies [58], AFM [63], and constricted migration assays [64].

Despite these significant contributions of the cytoskeleton to nuclear integrity, a majority of mechanical models consider only isolated nuclei, in which vimentin does not contribute to nuclear mechanics [8]. The models that consider the cytoskeleton often seek to model its role as either a compressive element in nuclear flattening [36,37,49,50] or a mechanism of facilitating constricted migration [39,41]. Only one mechanical model has studied the role of vimentin in AFM simulation; vimentin was modeled as a cage-like structure and simply shown to resist nuclear deformations [49]. There is then a need in the current literature surrounding mechanical models that include the cytoskeleton as a relevant structure to elucidate its specific role in either inducing or protecting nuclear strain and deformation.

### 3.2. Lamins

The second mechanical constituent of the nucleus is the nuclear lamina. Here, we provide a review of the nuclear lamina as well as the ways it has been mechanically modeled. The nuclear lamina forms a thin protein meshwork along the inside of the nuclear envelope and consists of two primary types: A- and B-type lamins [65]. A-type lamins consist of lamin A and C; B-type lamins consist of lamin B1 and B2. The nuclear lamina is of particular interest in nuclear modeling because it is a major mechanical component of the nucleus [66] and thus has roles in nuclear morphology [37], bleb formation [65], and nuclear rupture [67], especially during migration [68,69,70]. Early micropipette aspiration experiments showed the relevance of the nuclear lamina to the elastic response of nuclei [71]. Lamins’ storied role in nuclear mechanics has led to its inclusion in most mechanical models. Most commonly (and simply), the nuclear lamina has been modeled as a linear elastic shell, either infinitely thin [7] or with some finite thickness [16,18,20,35,36,38]. This serves primarily to capture the mechanical resistance from stretching of the nuclear lamina, but may oversimplify key experimental findings. Lamin B1 has itself been experimentally shown to be strain stiffening [72]. Along these lines, lamin A/C and B1/B2 have been shown to have a low persistence length suggesting they are easy to bend but hard to stretch like idealized worm like chain models [23]. Simulations using a hyperelastic shell model for the nuclear lamina employed by some investigators [39,48] are then more applicable for modeling this behavior as it captures the nonlinear force response at high strains. There is further experimental evidence that the nuclear lamina has a viscous response on relevant timescales [73,74,75]; various groups have sought to model this by treating the nuclear lamina as a viscoelastic material as opposed to purely elastic [41,44]. Outside of continuum theory, the lamina has been modeled as a meshwork of polymers [8,26,27,42,49], providing a more physical representation of the true structure visualized and detailed by super-resolution microscopy studies [21,22] and cryo-electron tomography [23]. Such modeling allows for studying the implications of altered mesh sizes, fiber stiffness, and bond strengths, which are parameters not easily accessible through experimentation or continuum modeling. Because of its clearly demonstrated importance in nuclear integrity, the nuclear lamina is an essential component of any nuclear mechanical model.

The majority of the previously mentioned techniques of modeling the nuclear lamina treat it as a single-material system; this is not the case. As previously noted, the nuclear lamina consists of both A- and B-type lamins, which serve distinct roles both in nuclear mechanics and nuclear function. It was originally thought that only lamin A/C was relevant for nuclear mechanics [76], and that lamin B1 was more so associated with proper orientation of the nucleus relative to the cell body [77]. However, it was later shown in micropipette aspiration experiments that the ratio of A-type to B-type lamins was positively correlated with nuclear stiffness [74]. Additional work has shown that when nuclei with already low levels of lamin A/C are experimentally depleted of lamin B1, nuclei become stiffer in both micropipette aspiration [78] and micromanipulation experiments [8]. Furthermore, viscoelastic studies have suggested that lamin A/C could be the primary viscous contribution and lamin B1/B2 could be the primary elastic contribution to mechanical response [74,79]. Although, there is no mechanical modeling to back these conclusions. Recent work has also shown that lamin B2 mimics the role of lamin A/C in that decreasing levels of lamin B2 induces nuclear softening and increases migration [80]. The distinctions between lamin A and C as well as lamin B1 and B2 provide an additional level of depth to be researched. Not only do they serve separate mechanical purposes, but they also are post-translationally modified differentially and spatially separated as well. While both lamin A and B1/B2 are post-translationally farnesylated, lamin A is further processed to lose the farnesyl group and lamin B1/B2 maintains it [81]. This differential farnesylation is believed to underlie the reason lamin B1/B2 resides approximately 10–20 nm closer to the nuclear periphery as determined by super resolution microscopy methods [82]. To date, few models have sought to account for the different types of lamins. A polymer-based model [42] and a CM model [43] have worked to capture this by treating lamin A/C and lamin B1/B2 as distinct materials to study bleb formation. However, there are little-to-no mechanical models that treat lamin A/C and lamin B1/B2 as separate materials with different viscoelastic properties in the common assays such as AFM and micropipette aspiration. For a complete model of the complexity of the nuclear lamina, one would need to account for each trait detailed here—elasticity, strain stiffening, viscosity, polymeric structure, separation of lamin A/C and lamin B1/B2—as well as the variation in density of lamin A/C and lamin B1/B2 around the nuclear surface.

### 3.3. Chromatin

Chromatin is the final mechanical constituent of the nucleus. Initial micropipette aspiration studies of isolated *Xenopus* oocyte nuclei concluded that chromatin had little role in the mechanical properties of nuclei [71]. Later research, however, has concluded otherwise, specifically showing that the compaction levels of chromatin are directly related to nuclear stiffness and dominate small deformations [7,8,15,75,83,84,85]. Early models of nuclear mechanics consistently refer to the nuclear interior as the “nucleoplasm” [16,20]; this term inaccurately portrays the nuclear interior as purely fluid-like. Experimentally, the chromatin has been observed to have an elastic response which is inconsistent with pure fluid-like behavior. The simplest model of the chromatin is then a purely elastic solid, which captures only the elastic response and ignores the viscous contribution from the surrounding fluid [7,49]. An improved approach is taken by other groups where the chromatin and the surrounding fluid are more appropriately modeled as viscoelastic [16,20,35,41], which is consistent with experimental observations in intranuclear protein mobility [86] and micropipette aspiration [9,10,87]. A further alternative approach is the poroelastic model of chromatin [17,39]. A poroelastic material accounts for viscous flow through defined pore sizes, similar to flow of the surrounding medium through the chromatin. Such materials are characterized by a strain-dependent viscous response, which has been observed in AFM studies [17]. This builds on purely viscoelastic treatments as it can inform how the compaction state of chromatin, and the subsequently altered pore size could have an influence on the viscous response as well as the elastic response. Increased viscosity has also been shown to be a signature of high-risk leukemia cells [88]. These viscous contributions from bulk nuclear deformation must be carefully considered as they may not be present for physiologically relevant timescales of approximately 10 s of nm/s [89,90]. Outside of continuum theory, the chromatin has been modeled as a confined polymeric system tethered to itself and the nuclear surface [26,27]. With the recent developments regarding the mechanical role of chromatin, it is crucial that it be accurately modeled in studies of nuclear mechanics moving forward.

## 4. Modeling of Assays for Studying Nuclear Mechanics

One purpose of mechanical modelling is informing how changes in nuclear mechanics can be measured by experimental techniques. Most experimental studies use a simple schematic model to extract material properties from a given dataset. This approach can be quite useful in understanding the dominant response in a given system, yet it glosses over the intricacies of each assay. Additional computational modeling provides this added insight of the assays used to probe nuclear mechanical properties. Such modeling has been done for almost all experimental techniques, including AFM [7,16,91], micropipette aspiration [20], micromanipulation [8,27], constricted migration [38,39,41], substrate stretching [37], plate compression [19,35], and magnetic bead twisting [92]. A majority of these computational models are CM models because of the ease of defining a geometry consistent with the assay. Results of these models often show discrepancies from their schematic counterparts, generally providing a more accurate representation of the experimental data. 

### 4.1. Modeling Resolves Contrasting Experimental Results Across Assays

Each assay mechanically probes the nucleus in different ways; mechanical models are especially useful in determining how a given assay may be more or less sensitive to specific nuclear structures, time scales, or length scales. For example, one group developed nearly identical CM models of micropipette aspiration and AFM with conical tips, both modeling an isolated nucleus consisting of the outer and inner nuclear membranes, the nuclear lamina, and the “nucleoplasm” [16,20]. Their simulations showed that micropipette aspiration is highly sensitive to changes in the stiffness of the nuclear lamina relative to the “nucleoplasm” [20]. Their AFM simulations, however, were quite sensitive to changes in the elasticity of the “nucleoplasm” [16], or more appropriately the chromatin filling the nucleus. This provides insight into why early micropipette aspiration measurements did not see chromatin as a relevant mechanical constituent [71] until the chromatin was condensed drastically via divalent ions [75], whereas AFM studies have clearly shown the relevance of chromatin to the elastic response of nuclei [7,15]. These simulations showed that the intranuclear strain is dependent on the geometry by which the nucleus is deformed, which subsequently leads to more specified probing of the lamina in micropipette aspiration. Similar geometry dependence has been shown for simulated micromanipulation [27]. This highlights further how and specifically why conclusions made with one assay may not necessarily transpire directly to another assay. Such comparative modelling of assays could help clarify the seemingly conflicting experimental results of the mechanical role of actin previously noted where clear differences are seen between extensional and compressive measurements [8,58,59]. Mechanical models are paramount for providing more informed conclusions about one’s experimental data and are likely to clarify the origin of conflicting results as different perspectives rather than right versus wrong.

### 4.2. Emergent Mechanical Phenomena from Complementary Experimental Assays—Strain Stiffening

Models of nuclear mechanics assays have also been useful in explaining emergent experimental findings. A specific example regards the relative contributions of the nuclear lamina and chromatin. We previously described how both the nuclear lamina and chromatin contribute to the mechanical response of nuclei. Micromanipulation experiments of isolated nuclei were able to separate their respective roles. They showed the existence of a two-regime force response where the low-strain regime (<3 μm) is dominated by chromatin and the high-strain regime (>3 μm), specifically strain stiffening, was dominated by the nuclear lamina. The investigators developed an MD simulation of a polymer shell (lamina) filled with a cross-linked polymeric interior (chromatin) that recapitulated the experimental results of both strain stiffening as well as predicted buckling of the lamina in the absence of chromatin [8,27]. This work was built upon by a recent study showing similar strain-stiffening results in AFM compression which was validated by an CM model of an elastic solid surround by an elastic shell under AFM indentation [7]. In both instances, the mechanical models explained that geometry alone is sufficient to induce strain stiffening; non-linear material properties are not necessary. They additionally provided a means of simulating the effects of knocking down lamin A/C as well as decompacting chromatin, providing support to the conclusions that chromatin dominates small nuclear strains while the lamina provides strain stiffening at large nuclear strains. Finally, lamin strain-stiffening is supported by many experiments including in vitro filament stretching [72], short persistence length measurements [23], nuclear morphology during cell seeding [50], nuclear stress stiffening [75], and non-linear nuclear osmotic properties [93]. Observing emergent phenomena in nuclear mechanics must be solidified and backed by accurate mechanical models that validate the source of these phenomena. Here, lamin-based nuclear strain stiffening has been supported both by complementary experimental techniques of extension (micromanipulation) and compression (AFM), but also by complementary MD simulations and CM modeling, respectively.

## 5. Applications of Mechanical Models to Biologically Relevant Processes

Mechanical models have historically been useful in informing our collective understanding of a variety of biological processes. Here, we review the major topics for which these models have proven useful for understanding key biological processes. These topics include cellular migration, nuclear blebbing, nuclear rupture, and cell spreading and detachment. Specifically, we detail the major experimental observations and conclusions as well as how mechanical models have been used to explain these conclusions and predict phenomena to be observed in future experiments.

### 5.1. Constricted Cellular Migration

During processes such as cancer metastasis and immune response, cells migrate through tight constrictions resulting in extreme levels of strain, where lamin mechanics dominate. The cell nucleus—and more specifically the nuclear lamina stiffness—was experimentally shown to provide the rate-limiting step in such migration [79,94]. Softer nuclei, due to lower levels of lamin A/C, have been previously correlated to increased migration efficiency, potentially providing a connection because successful metastasis and altered mechanical properties [79]. Alternatively, recent experimental work has shown also that nuclei treated with trichostatin A (TSA) to decondense chromatin and subsequently induce nuclear softening leads to slower migration rates [95] but increased successful constricted migration [40]. This is not immediately intuitive as we traditionally understand migration speed to correlate with successful migration. However, decreased compaction could limit the ability of the nucleus to propagate the force necessary to traverse a constriction [96]. It is additionally known that active processes condense chromatin at the induction of cell migration [97]. Finally, the significant external stress on the nucleus during migration has experimentally been shown to cause nuclear rupture and subsequent DNA damage [68,69,70] as well as plastic deformation [39,79]. The physiological relevance of constricted migration combined with the role of mechanical properties in its efficiency make it well suited to be studied by mechanical modeling.

Both mechanical [38,41] and chemomechanical [39] models have been developed to better understand constricted migration. A crucial result shown in simulation is that decreasing the stiffness of the nuclear lamina allows for increased migration rates, which is consistent with the previously mentioned experimental data. While this does not appear immediately profound, this modeling result highlights that it is actually the mechanical properties of the nuclear lamina that limit migration. While this does not preclude a role for a downstream active response to lowered levels of lamin A/C, this shows that the mechanical properties alone are sufficient to explain the experimental observations. Similarly, modeling concludes that lower nuclear lamina stiffness is sufficient to increase nuclear plasticity [39,41]. Recent modeling work has begun to study the role of chromatin’s material properties on migration, showing through simulation that reducing the stiffness of chromatin decreases the amount of force the cell must generate to enter a constriction [40]. No modeling, however, has studied why chromatin decompaction serves to slow constricted migration speed. Some modeling has been done to understand the feedback mechanisms that regulate nuclear morphology and chromatin condensation levels [49]; however, no such modeling has been applied to constricted migration. Additionally, these models allow for predictions to be made; one such example is that nuclei undergo volume loss during constricted migration [39], which could have significant implications regarding chromosome territories and transcription as nuclear volume loss has been experimentally shown to lead the nucleus into a transcriptionally quiescent state [98]. While this has yet to be observed in migration assays, nuclear volume loss has recently been shown for similar magnitudes of compression in AFM experiments [7] and plate-compression assays [98]. Mechanical modeling of constricted migration has been crucial for confirming that nuclear mechanical properties in part govern successful migration, but further simulations could inform additional subtleties regarding the role of chromatin.

### 5.2. Nuclear Blebbing and Rupture

Nuclear blebs are defined to be abnormal protrusions from the nuclear surface. Formation of such blebs has been a prominent topic of recent research as they are often associated with a variety of disease states such as leukemia [99], prostate cancer [3], cervical cancer [4], breast cancer [5,6], progeria/advanced aging, and muscular dystrophy [2]. Depletion or mutation of lamins were the first and most prominent experimental changes that cause nuclear blebbing and ruptures. There is conflicting research regarding the composition of the nuclear lamina within nuclear blebs. Early work had defined blebs to be enriched only with lamin A/C and lacking of lamin B1/B2 [65]. However, a recent study has shown the existence of a bimodal distribution wherein approximately half of the blebbed population lacks lamin B1/B2 in the bleb, while the other half has lamin B1/B2 in the bleb [100]. The rate of bleb formation has been experimentally shown to increase with lamin B1 deficiencies [101]; recent research has also shown that nuclear blebs can proceed solely from chromatin decompaction and weakened chromatin-based nuclear mechanics, independent of changes to lamins [84,100,102]. Further experimental work has shown that the increase in entropic pressure from chromatin decompaction is sufficient not only to induce blebs, but to rupture nuclei [103,104]. Little is known, however, on the exact mechanism by which blebs form. Given their association with disease and nuclear rupture, bleb formation and stability are relevant phenomena to investigate through mechanical modeling. 

To study nuclear blebs through mechanical models, investigators have modeled the nuclear lamina as a two-material system, allowing them to separate the roles of A- and B-type lamins in bleb formation [42,43]. In the earlier study, A- and B-type lamins were treated as separate polymeric systems tethered together by a series of connectors. The investigators were more interested in understanding dynamics of blebs as opposed to mechanisms of formation. Blebs consisting of either both A- and B-type lamins or just A-type lamins were artificially induced with no physiological mechanisms prescribed. It was shown that blebs enriched in just A-type lamins were more mechanically stable, suggesting that the physical separation of A- and B-type lamins and the strength of their connections govern the stability of blebs. This model is also consistent with the reduction in αII-spectrin causing nuclear blebs of only one component [105]. The latter model used a CM approach wherein they modeled the nuclear lamina as a shell with each spatial location corresponding to a location of enriched in either A- or B-type lamins [43]. They modeled bleb formation by considering the preferred nuclear shape due to minimization of bending and stretching energy as a function of the relative amount of A-type lamins to B-type lamins as well as the mesh sizes of each structure. It was assumed in the model that A- and B-type lamins have different preferred curvatures and mesh sizes. Their model showed that a difference in mesh sizes between A- and B-type lamins was necessary for forming a blebbed system. Both of these models of nuclear blebs predated the recent research regarding the role of chromatin in bleb formation; neither model considered chromatin in their simulations. While these models have provided solid initial steps in understanding the mechanics of blebs, they do not exactly recapitulate experimental findings. A recent model, however, has considered chromatin’s role in bleb formation [26]. They showed specifically through simulation that tethering of the chromatin to the nuclear lamina at lamin-associated domains was necessary to induce blebbing. However, this model does not separate A- and B-type lamins and therefore cannot shed light on their roles in bleb formation. There is then room to build upon these works to further understand the mechanisms by which blebs form and their mechanical properties.

The formation of blebs can often lead to nuclear rupture, which is believed to be associated with nuclear dysfunction. Such rupture events are often associated with cellular migration [68,69,70], but it also is known to occur as a result of actin-based confinement [55]. Furthermore, recent work has shown that local tensile [46] and compressive forces [106] are sufficient to rupture nuclei as well. Such rupture events provide a chance for cytoplasmic contents to mislocalize into the nucleus, and vice versa [107]. With flow across the nuclear envelope no longer regulated by the generally selective nuclear pore complex [108], a variety of groups have observed an increase in double-stand DNA breaks [68,69,70,109]. This has obvious implications for proper nuclear and cellular function, and is worthy of deeper mechanical analysis and simulations to understand the basis. 

Nuclear rupture itself has also been the subject of recent mechanical models [44,45,46]. One analytical approach considers two limiting cases where the nuclear interior is treated either as a semi-flexible polymer that forms a channel to the rupture site or as a simple viscous fluid. Interestingly, their conclusions regarding the dynamics of the rupture hole were qualitatively similar for both cases. Their model showed that upon rupture, the size of the hole increases exponentially to a maximal radius before closing linearly in time. Furthermore, their model concluded both that chromatin herniations are exponentially sensitive to the radius of the rupture site, and that increased viscosity of the nuclear lamina reduces this rupture radius thus minimizing chromatin herniation [44]. This highlights that the material properties of the nuclear lamina are relevant for limiting the size of the rupture site and subsequently the magnitude of the chromatin herniation. Building on this earlier work, a second analytically solved CM model was developed to study nucleation mechanisms of blebs in the nuclear lamina. They used an energetics approach to study how the inclusion of nuclear pore complexes (NPCs) alters the scaling relationships between hole nucleation rate and strain in the lamina. They were able to predict that increasing the density of the lamina induces a transition from homogeneous nucleation (no NPCs) to heterogeneous nucleation (with NPCs), which could be validated with further experiments. A separate CM model of diffusion of EGFP-NLS at local nuclear rupture sites has helped in demonstrating that the magnitude of a local nuclear stress is directly related to the size of the rupture size [46], which subsequently governs the rate of mislocalization of nuclear contents to the cytoplasm. Finally, a recent model sought to distinguish whether the DNA damage associated with nuclear rupture and specifically constricted migration is due to mechanical stress or could be explained by separation of repair proteins from the chromatin. Through treating the nucleus as an elastic-fluid system wherein the fluid surrounding the chromatin can be squeezed out of the nucleus, their model showed that outflow of mobile repair proteins due to the constricted migration was sufficient to explain the experimental data on increased damage sites [47]. Recent experimental work has shown similarly that nuclear deformation alone can cause increased DNA damage [110]. In their model, nuclear rupture was assumed to merely delay the ability of the repair factors to return to their original locations. This profound result infers that mechanically induced separation of repair factors from damage sites could be more important for increases in DNA damage than the actual mechanical stresses themselves. It is clear then that mechanical models have shaped the collective understanding of the dynamics and causes of nuclear rupture and DNA damage, specifically that the scale of the rupture site is dependent on the material properties of the lamina which has consequences for the amount outflow of nuclear contents into the cytoplasm. Although, chromatin and its mechanical properties also influence nuclear rupture and DNA damage [111]. Additional mechanical modeling is needed along with experiments to fully understand how nuclear rupture occurs and effects nuclear functions and leads to DNA damage. 

### 5.3. Cell Spreading and Detachment

As cells adhere to substrates, they begin to spread and subsequently flatten nuclei; the stiffness of the underlying substrate regulates how much cells are able to spread [112]. Experimental data shows both that nuclear height and surface roughness are reduced in spread cells; lamin A/C deficiencies subsequently re-introduce nuclear surface roughness in spread cells [37]. This nuclear flattening has also been shown to be dependent on the geometry upon which cells can spread [98], and recent experimental work suggests that the movement of cell boundaries is sufficient to shape the nucleus during this process [113,114]. Additionally, it was observed that upon removal from the cell body, nuclear shape was unchanged [114]. Such permanent changes to cell geometry ultimately alter chromatin organization and subsequently cellular function [115]. 

Several mechanical models have been constructed to understand how nuclear morphology changes as cell adhere or detach from substrates [48,50] and how cell geometric constraints alter nuclear morphology as well [49]. In an analytical model presuming the nucleus to be a pressurized sphere with an elastic shell under uniform plate compression representative of the actin cap [37], investigators derived equations linking nuclear volume and surface area to the magnitude of external force and the elastic modulus [36]. Although, this model clearly neglects the previously detailed role of the chromatin in this process. In one simulation, the nuclear lamina is treated as a neo-Hookean (hyperelastic) material of finite thickness and there existed a pressure differences across the nuclear envelope. Their model showed that nuclei undergo significant volume loss and wrinkling after detachment consistent with experimentation. Both the volume loss and wrinkling were dependent on the thickness of the nuclear lamina and the magnitude of the pressure gradient. This highlights the role of the nuclear lamina and pressure in regulating nuclear shape. A separate study focused more on nuclear morphology during cell spreading [50]. It was shown that in general, nuclei extend their surface area during spreading up until it begins to stretch; the volume remained mostly constant during the spreading process. This is consistent with a strain-stiffening response that results from the lamina becoming taut and stretching [7,8]. Their simulations showed how nuclear flattening could occur without actomyosin activity or bundles, microtubules, the linker of the nucleoskeleton and cytoskeleton (LINC) complex, or intermediate filaments; this is consistent with the experimental observations claiming that the movement of cell boundaries is sufficient to shape the nucleus. Finally, a recent model has sought to study the feedback mechanisms between cell adhesions, the cytoskeleton, and the nucleus [49]. Their model was able to show that this three-way feedback system recapitulated the experimental results of the dependence of nuclear flattening on cell geometry. Cell spreading is a phenomenon often studied purely through observation; mechanical modeling has been fundamental in explaining the roles of cell boundaries and pressure in the manner by which the nucleus is shaped during this process.

## 6. Outlook on Mechanical Models

Although investigators have been mechanically modeling cell nuclei for over fifteen years, there are still several open areas of research where models could be useful. In this concluding section, we outline several facets of nuclear mechanics where sufficient mechanical models are lacking. These topics include the roles of tethers between chromatin and the nuclear lamina, the role of links between the cytoskeleton and the nucleus, and the separation of A- and B-type lamins. A myriad of intricate connections exists between the chromatin, lamina, and the cytoskeleton; we have focused our description on a subset of these connections we feel to be of particular biophysical relevance. Additional problems warrant further studies and modeling, specifically how cell type and mechanosensation of the environment may modulate nuclear mechanical properties. However, we have chosen to focus this section on connections between the nuclear mechanical constituents as opposed the aforementioned additional considerations. Each of these areas of research prove to be relevant for understanding laminopathies and cellular function; mechanical modeling then can inform a more complete understanding of these intricacies.

### 6.1. Lamin–Chromatin Connections

As previously described, the nuclear lamina and the chromatin are the two dominant nuclear structures regarding mechanical stability. The chromatin and nuclear periphery are mechanically tethered together; this tethering has been experimentally shown to be relevant for nuclear stability [116]. In mechanical modeling, such tethering could be presented as a boundary condition between the chromatin and nuclear lamina in CM models or as a physical link in MD simulations. A majority of CM models assume this boundary condition to be a no-slip condition, meaning relative motion of the chromatin and lamina at the boundary is not allowed. It is unclear, however, how valid this assumption is as laminopathies and lamin deficiencies disrupt lamin-associated domains (LADs) [117,118] and potentially invalidate such assumptions. One recent study has begun to investigate such questions with mechanical modeling [26]. Their mechanical model required that lamin–chromatin tethers are localized along lamin domain boundaries to form nuclear blebs and abnormal nuclear morphologies. Additionally, these connections are necessary for modeling strain stiffening in micromanipulation [27]. There is little-to-no work, however, on how such variations in the boundary conditions may present itself in common assays such as AFM or micropipette aspiration. For example, an experimental micropipette aspiration study observed an increase in chromatin mobility when lamin A/C was knocked down in human A549 cells [75]; distinguishing whether this is, in part, due to a reduction in tethering of the chromatin to the nuclear lamina could be achieved through mechanical modeling with varied boundary conditions. Mechanical models with tunable chromatin–lamin tethering could then prove highly useful for furthering our understanding of laminopathies.

### 6.2. Lamin–Cytoskeleton Connections

The second subsection of nuclear mechanics that is lacking sufficient modeling is in the role of mechanical links between the cytoskeleton and the nuclear lamina. Numerous proteins exist amongst these connections. One subset of these proteins is known formally as the linker of the nucleoskeleton and cytoskeleton (LINC) complex, and consists primarily of nesprins and SUN proteins that link that cytoskeleton to the outer nuclear membrane and the inner nuclear membrane to the nuclear lamina, respectively [119]. Experiments have demonstrated the importance of the LINC complex for transferring mechanical signals from the cell surface to chromatin [31,120], which subsequently can lead to altered transcriptional activity due to chromatin stretching [32]. Laminopathies have been shown to disrupt this connection [121,122], which subsequently alters a cell’s ability to process mechanical signals. Models of the cytoskeleton have predominantly focused on the cytoskeleton as a means of facilitating cellular migration [39,41]; little work, however, has sought to model the mechanical implications of disrupting these connections. Effective models could then inform how laminopathies alter nuclear mechanics and mechanotransduction.

### 6.3. Separate But Interacting A-type vs. B-type lamin Meshworks

The final area well-suited for additional modeling is the distinction of A- and B-type lamins in the mechanical response of cell nuclei. As discussed previously, A- and B-type lamins serve distinct mechanical roles. More specifically, it has been experimentally shown that decreasing expression of lamin B1 and increasing expression of lamin A/C both result in nuclear stiffening; this implies the ratio of lamin A/C to lamin B1 to be a proper metric of nuclear stiffness [8,78], in agreement with landmark initial findings [74]. Additionally, experiments suggest that lamin A may govern the viscous response, while lamin B could dictate the elastic response [74,79]. However, only mechanical models of nuclear blebbing have sought to distinguish A- and B-type lamins [42,43]. There are no detailed mechanical models to date that explicitly explore these separate roles for common force measurement assays such as AFM, micromanipulation, or micropipette aspiration. Such models would inform our understanding of why the experimental results detailed above have been observed. Given the myriad of diseases associated with mutations in the nuclear lamina and the clearly made experimental distinctions between the isoforms, to not begin distinguishing between A- and B-type lamins in mechanical models would be to place fundamental limitation on the intersection between mechanics and disease.

## 7. Conclusions

The role of the cell nucleus is multi-faceted; it both houses the entire genome and regulates cellular activity as well as provides mechanical support to the cell. These roles, however, are inherently coupled as the mechanical properties and integrity of the nucleus facilitate how the nucleus interprets mechanical stimuli, which can subsequently alter a cell’s response to its environment. Laminopathies and other disease states disrupt the mechanical integrity of the nucleus, causing nuclear softening, increased nuclear blebbing and rupture, spikes in DNA damage, and broken mechanotransduction pathways. Because of the role of mechanical properties in these disease-associated phenomena, mechanical modeling serves a vital role in cultivating our understanding of these phenomena. Models generally fall into being schematic or nucleus-specific: the former consists mainly of a spring–dashpot system used to fit experimental data, while the latter is dominated by analytically or computationally solved CM models and MD simulations. These models have proven useful for studying not only assays aimed to measure mechanical properties of nuclei, but also for explaining emergent phenomena such as nuclear strain stiffening. Models have the capacity to not only explain results, but also make predictions about behaviors that are yet to be observed. This aspect of modeling is currently under-utilized in the field and could serve a vital role in guiding experimentation. Additionally, biologically relevant phenomena ranging from constricted migration to cell spreading are well informed by sufficient modeling. Despite the fact that mechanical modeling of cell nuclei has been underway for over 15 years, the field remains relatively young. Each biologically relevant process discussed in this review features, at maximum, a handful of models; there are then a myriad of open questions that can be investigated through proper modeling. We specifically outlined three areas of research—namely connections between chromatin and lamins, connections between lamins and the cytoskeleton, and distinctions between A- and B-type lamins—for which mechanical modeling is desperately needed. Effective models that account for the role of chromatin and its connections to lamins, distinctions between A- and B-type lamins, the LINC complex, and the protective and antagonistic role of the cytoskeleton would serve to dramatically improve our collective understanding of the role of the nucleus in disease.

## Figures and Tables

**Table 1:** A summary of the primary advantages and limitations of the three classes of models.

Model Type	Advantages	Limitations
Schematic	Easily solved analytically Provides a simple equation or set of equations to fit to a given dataset Effective at detecting global changes in mechanical properties Effective at studying limiting cases where certain structures dominate the system’s response	Not specific to nuclei, but rather applied to nuclear mechanics data Require unrealistic assumptions regarding homogeneity, dimensionality, and geometry (1D) Limited in their ability to separate contributions of specific nuclear structures
Continuum Mechanics (CM)	Can be solved either computationally or analytically Allow for realistic nuclear geometries Ability to prescribe different mechanical properties to each structure being modeled Successful at studying assay-specific nuclear deformations	Assume each material to be continuous, thus limiting the ability to model polymeric structures or variations in protein concentration Mechanical properties for each material are prescribed a priori as opposed to being emergent
Molecular Dynamics (MD)	Quasi molecular-scale modeling of nuclear constituents gives a more accurate representation of the polymeric structures Ability to prescribe strength and number of bonds in a given material and between materials Global material properties are emergent from local, molecular interactions	Computationally intensive due to quasi molecular-scale modeling Accurate knowledge of interactions between monomers is required Prescribing complex geometries is more difficult than in CM models

**Table 2:** Summary of nucleus-specific mechanical models. Column 1 additionally provides the class of model. CM-A: analytically-solved continuum mechanics model. CM-C: computationally-solved continuum mechanics model. MD: molecular dynamics model.

Assay or Phenomenon	Cytoskeletal Component	Lamin Component	Chromatin Component	Simulation Conclusions	Reference
AFM (CM-C)	N/A	Elastic Shell	Elastic Solid	Nuclei exhibit strain stiffening in AFM Chromatin resists volume changes at small indentations Lamin A/C resists surface area changes causing strain stiffening at large indentations	[7]
AFM (CM-C)	N/A	Elastic Shell	Viscoelastic Solid	Including the nuclear envelope is necessary to recapitulate the magnitude and shape of experimental force versus indentation curves on cells Force response is sensitive to elasticity of “nucleoplasm” Force response is highly dependent on probe angle	[16]
AFM (CM-C)	N/A	N/A	Poroelastic	Nuclei exhibit depth-dependent relaxation rates, consistent with poroelastic materials	[17]
AFM (CM-C)	N/A	Elastic Shell	Elastic Solid (radially decaying Elastic Modulus)	A radially decaying elastic modulus recapitulates experimentally determined depth-dependent elastic moduli Presence of nuclear lamina adds an overall increase in stiffness	[18]
Micropipette Aspiration (CM-C)	N/A	Elastic Shell	Viscoelastic Solid	Micropipette aspiration measurements are highly sensitive to the stiffness of the nuclear lamina	[20]
Micromanipulation (MD)	N/A	Polymer Shell	Confined Polymer	Nuclear lamina buckles with lack of chromatin Chromatin provides the short extension force response Lamin determines strain stiffening due to the geometry of the nucleus during long extension Two regime force response requires both chromatin–chromatin and chromatin–lamin tethers	[27] [8]
Plate Compression (CM-C)	N/A	Elastic Shell	Viscoelastoplastic	Lower stiffness of the nuclear lamina increases nuclear plasticity Increased stiffness of the “nucleoplasm” increases nuclear plasticity	[35]
Plate Compression (CM-C)	Hyperelastic	N/A	Hyperelastic	Force response is dependent on cell and nuclear geometry, with spread cells appearing stiffer than round cells, both of which appear stiffer than isolated nuclei	[19]
Actin Compression (CM-A)	Uniform compressive plate	Elastic Shell	N/A	Provide equations linking nuclear shape to applied force and elastic modulus	[36]
Substrate Stretching and the Actin Cap (CM-C)	Shear deformable beams	N/A	Elastic Solid	Stress concentrates along the edges of the nucleus in absence of actin cap Absence of actin cap increases nuclear stress	[37]
Constricted Migration (CM-A)	N/A	(i) Elastic Shell (ii) N/A	(i) Inviscid Fluid (ii) Elastic Solid	Provide relationship between mechanical properties and active processes for migration There exists a critical pore radius for which a cell can enter based upon nucleus stiffness and the ability to form adhesions	[38]
Constricted Migration (CM-C)	Driving force for migration	Hyperelastic Shell	Poroelastic	Resistance to transmigration is dependent on extracellular matrix (ECM) stiffness, pore size, and lamin A/C stiffness Lower lamin A/C stiffness results in increased nuclear plastic damage Model predicts buckling of the lamina, nuclear rupture, and volume loss	[39]
Constricted Migration (CM-C)	N/A	N/A	Hyperelastic Solid	There exists a critical force a cell must overcome to enter a constricted pore The critical force increases as the pore size decreases and/or the stiffness of the environment increases Decreases in the stiffness of the nucleus decrease the critical force	[40]
Constricted Migration (CM-C)	Driving force for migration, viscoelastic	Viscoelastic Shell	Elastoplastic	Nuclear softening increases invasiveness Nuclear stiffening increases plastic damage of the nucleus Constricted migration leads to kinking of the nuclear membrane	[41]
Nuclear Blebbing (MD)	N/A	Two-Polymer Shell	N/A	Retraction of blebs with only A-type lamins follow a double-exponential decay Retraction of blebs with A- and B-type lamins follow an exponential decay One-component blebs can stabilize in the blebbed state	[42]
Shape Fluctuations and Nuclear Blebbing (MD)	Point particles connected to lamina via springs	Polymer Shell	Confined Polymer	Tethering between chromatin and nuclear lamina is necessary for bleb formation Stiffness of connection between the nucleus and cytoskeleton correlates with nuclear shape fluctuations	[26]
Nuclear Blebbing (CM-C)	N/A	Two-Material Elastic Shell	N/A	Larger mesh size of A-type lamins relative to B-type lamins is required to form nuclear blebs	[43]
Nuclear Rupture and Chromatin Herniation (CM-A)	N/A	Viscoelastic Shell	Semi-Flexible Polymer	Rupture site radius increases exponentially to a critical value before closing linearly in time Increased viscosity of the nuclear lamina minimizes rupture radius Chromatin herniations are exponentially sensitive to rupture radius	[44]
Nuclear Rupture (CM-A)	N/A	Elastic layer with and without nuclear pore complexes	N/A	Develops scaling laws between hole nucleation rate and strain on the lamina for homogenous and heterogeneous lamina layers Predicts that increased lamin density correlates with a transition from homogenous to heterogeneous nucleation mechanisms	[45]
Nuclear Rupture (CM-C)	Fluid	N/A	Fluid	Rate of outflow of nuclear contents correlates with the diameter of the rupture site	[46]
DNA Damage (CM-A)	N/A	N/A	Elastic-Fluid	Separation of repair proteins from the chromatin resulting in delayed repair is sufficient to recapitulate experimental observation of increased DNA damage in constricted migration	[47]
Cell Detachment and Attachment (CM-C)	Compressive plate	Hyperelastic Shell	N/A	Higher pressure and thinner nuclear lamina increase wrinkling of detached nuclei Nuclear volume decreases upon detachment	[48]
Cell Spreading, Geometric Constraints * (CM-C)	Provide compressive stress	Stiffening filamentous network	Elastic Solid	Cell geometry alters local stresses which regulate nuclear architecture and mechanics A 3-way feedback mechanism between the nucleus, the cytoskeleton, and adhesions recapitulates experimental results regarding cell geometric constraints and can predict implications of cytoskeletal disruptions	[49]
Cell Spreading (CM-C)	Compressive contractile network	Elastic Shell	Elastic Solid	Cell spreading is necessary and sufficient to drive nuclear flattening	[50]

* This work features several different models for which a full description is outside the scope of this table.
